# Inequality in outpatient resource utilization among older adults during the 2007–2008 financial crisis: findings from Taiwan

**DOI:** 10.1186/s12913-019-4466-6

**Published:** 2019-09-02

**Authors:** Chiao-Lee Chu, Yu-Hua Chu, Chih-Yuan Lin, Yen-Ping Hsieh, Ching-Sung Ho, Yung-Yu Su, Chia-Nien Liu

**Affiliations:** 1 Department of Long Term Care, National Quemoy University, No. 1, University Rd., Jinning township, Kinmen County, 89250 Taiwan, Republic of China; 2 School of Dentistry, National Defense Medical Center, No.161, Sec. 6, Minquan E. Rd., Neihu Dist., Taipei City, 11490 Taiwan, Republic of China; 3 Department of Dental Technology and Materials Science, Central Taiwan University of Science and Technology, No.666, Buzih Road, Beitun District, Taichung City, 40601 Taiwan, Republic of China; 4 Department of Senior Citizen Welfare and Long-term Care Business (master program), Hungkuang University, No. 1018, Sec. 6, Taiwan Boulevard, Shalu District, Taichung City, 43302 Taiwan, Republic of China; 5 Department of Living Science, National Open University, No.172, Zhongzheng Rd., Luzhou Dist.,, New Taipei City, 247 Taiwan, Republic of China

**Keywords:** Medical care utilization, Concentration index, Health inequality, Economic crisis, Older adults

## Abstract

**Background:**

Equity in medical resource utilization is a crucial concern in countries with national health insurance systems. From the perspective of an active aging framework, public health insurance is one of the pillars of economic security, as suggested by the World Bank, to achieve the goal of social security for older adults. This study thus sought to quantify income-related inequality in national health insurance systems, especially during the global economic crisis of 2007–2008.

**Methods:**

By employing the Taiwan National Health Interview Surveys (2005 and 2009) datasets, we analyzed the socioeconomic inequality of outpatient service utilization for older Taiwanese adults during the financial crisis of 2007–2008. We used corrected concentration indices (CCIs) to examine inequalities over time. Furthermore, we decomposed socioeconomic inequalities to reveal the contributions of determinant factors. The dependent variables related to whether participants had used outpatient services in the previous month, and the independent factors included individual’s needs, enabling, predisposing, and environmental factors proposed by Andersen model.

**Results:**

The sample consisted of 2415 observations in 2005 and 2554 observations in 2009. The income-related health care inequality was minor from pro-rich to pro-poor across the study duration, although the difference was insignificant (women: from a concentration index [CI] of 0.0256 in 2005 to a CI of − 0.0098 in 2009; men: from a CI of 0.0379 in 2005 to a CI of 0.0310 in 2009). We used a probit model to analyze the effect of explanatory factors on outpatient resource utilization by men and women. After other factors were controlled for, we found that that the income variable had a positive and significant effect on outpatient service utilization in 2009 and the absolute contribution of income to inequality notably increased from 0.0480 in 2005 to 0.3247 in 2009 for older women.

**Conclusions:**

In conclusion, Taiwan’s National Health Insurance system guarantees the accessibility of health care services to older adults, but slight income-related inequality was maintained in the outpatient resource utilization of women during the 2007–2008 financial crisis. Close attention should be paid to the vulnerability of women during times of economic insecurity.

## Background

The World Health Organization has propagated its *Active Aging Policy Framework* to guide international efforts to address the challenges related to global aging [1]. The supply of public health insurance is a pillar of social security policy that supports active aging. Furthermore, public health insurance is a pillar of economic security mentioned in the World Bank’s social security policy for elderly people [2]. The aim of these aging policy frameworks is to enhance quality of life as people age. Similar to other advanced countries, Taiwan is facing challenges related to population aging. Taiwan’s aging rate is over twice that of the United States and some European countries. In 1993, Taiwan became an aging society (≥7% of people are aged 65 years or older), and it was declared to have an aged society (≥14% of people are aged 65 years or older) in April 2018 [3].

The Taiwanese government implemented its National Health Insurance (NHI) system in 1995, which is a single-payer, universal health insurance scheme. The scheme provides comprehensive health care to its users and is primarily financed through payroll contributions [4, 5]. Taiwan’s NHI was intended to remove economic barriers to medical resource utilization and provide all citizens (including elderly citizens) with equal access to health care. Studies have demonstrated that health care resource utilization and service quality for low- or middle-income groups increased after Taiwan’s NHI system was implemented [6, 7]. In other countries, similar conclusions have been reached regarding the relationship between national health insurance benefit coverage and income-related inequality in medical resource utilization [8]. Some studies have concluded that providing public health insurance can improve the equity of health care resource distribution.

A national health insurance system can protect older adults from financial crises resulting from medical resource utilization. However, few studies have quantified the degree of socioeconomic or income-related medical resource utilization inequality in national health insurance systems during a global financial crisis. This study aimed to quantify income-related inequalities and study the socioeconomic determinants of inequality during the global financial crisis of 2007–2008. This crisis, the most severe since the Great Depression, began with the subprime mortgage crisis in the United States in 2007. The impact of this global crisis resulted in downside risks to local economies, and Taiwan’s economy was severely affected by the financial crisis in the autumn of 2008, similar to other developing countries [9]. The approach taken in this study has crucial policy-related implications because it could improve knowledge related to the protective effect of Taiwan’s NHI on the health of older adults. Another contribution of this study relates to its analysis of the health care utilization of older adults during a financial crisis.

## Methods

### Material

Cross-sectional data for the period 2005–2009 were taken from the Taiwan National Health Interview Survey (NHIS). This representative survey of the population of Taiwan is conducted by the Health Promotion Administration of the Ministry of Health and Welfare, Taiwan. The NHIS employs a systematic, multistage, and stratified sampling design in which probability proportional to size sampling is applied. The degree of urbanization, geographic location, and administrative boundaries are controlled [10, 11]. In consideration of the purposes of this research, for our sample, we selected participants who were aged 65 years and older. We focused only on the outpatient visit utilization of elderly adults.

### Measuring and decomposing inequalities

By using the concentration index (CI) [12, 13], we measured and decomposed income-related inequality associated with outpatient resource utilization. The following equation was used to calculate the CI:

$$ \mathrm{CI}=\frac{2}{\overline{\mathrm{y}}}\operatorname{cov}\left({\mathrm{y}}_{\mathrm{i}},{\mathrm{R}}_{\mathrm{i}}\right) $$

where $\overline{\mathrm{y}}$ is the mean value of the ill-health proxy (that is, outpatient utilization), y_i_ is the ill-health of *i*th individual, and R_i_ is the cumulative percentage that each individual represents over the total population after population ranking based on income has been conducted. The values of the CI range from − 1 to 1. Prevalence of ill-health status in individuals with a relatively low (high) income is indicated by a negative (positive) CI value. If the CI value is 0, it indicates no income-related inequality in the distribution of ill-health status.

For cases where the ill-health variable is dichotomous, Erreygers proposed the corrected CI to deal with the bounded nature of binary nature of ill-health variables (i.e., between 0 and 1). This enables comparisons between individuals (and groups of individuals) that may present different ill-health statuses [14]. The equation for the corrected CI is as follows:

$$ \mathrm{E}\left(\mathrm{y}\right)=\frac{4\overline{\mathrm{y}}}{{\mathrm{y}}^{\mathrm{max}}-{\mathrm{y}}^{\mathrm{min}}}\mathrm{CI} $$

where y^min^ and y^max^ are the minimum and maximum value of the ill-health variable, respectively.

Using regression techniques, decomposition of the CI is possible to measure how different factors contribute to income-related inequality of outpatient resource utilization [15]. The decomposition of inequality can be shown as follows when Erreygers’ corrected index is used [16]:

$$ \mathrm{E}=4\sum \limits_{\mathrm{k}}\left({\upbeta}_{\mathrm{k}}^{\mathrm{m}}{\overline{\mathrm{x}}}_{\mathrm{k}}\right){\mathrm{CI}}_{\mathrm{k}}+\mathrm{GC}{\mathrm{I}}_{\upvarepsilon} $$

where E is the Erreygers corrected CI, ${\overline{\mathrm{x}}}_{\mathrm{k}}$ is the mean of explanatory variables, ${\upbeta}_{\mathrm{k}}^{\mathrm{m}}$ is the partial effects evaluated using sample means, CI_k_ is the CI mean, and GCI_ε_ is the generalized CI for the error term.

### Definition of variables

#### Inequality CI

For the measurement of inequality through a decomposition analysis and CI, a continuous variable is required that can rank individuals according to their socioeconomic statuses. This was problematic in the present study because the NHIS only recorded the monthly income data of the sample group as a categorical variable with 10 response intervals for 2005 and 2009. We were thus first required to select a variable from the aforementioned income categories for continuous ranking. A continuous variable for income was estimated from the calculated predictions of an interval regression model according to the demographic data of the participants, with covariates including age, age-squared, education level, previous working status, and mean household income for the region of residence. The income of elderly men and women in 2005 and 2009 was separately computed using this method. Because the level of income would not be less than NT$1, we set the monthly income for older adults to be more than NT$1. Furthermore, the modified Organization for Economic Co-operation and Development (OECD) equivalence scale was adopted to calculate equivalent income, and by using the consumer price indices provided by the Directorate General of Budget, Accounting, and Statistics of the Executive Yuan, the equivalent income was rescaled to the price level of 2009.

#### The dependent variable

We used the question “Have you used outpatient services in the last month?” from the NHIS questionnaire as the dependent variable. Participants responded “yes” or “no” to this question. This variable is a dichotomous (binary) dependent variable.

#### Explanatory variables

The demand for health care services demand depends on user needs as well as enabling, predisposing, and environmental factors [17]. Needs are related to an individual’s health status, and enabling factors are those that influence the use of health care resources, such as place of residence, income level, and health insurance [18, 19]. Predisposing factors include health beliefs and demographic characteristics, and environmental factors include the health care system and access to health care services, such as the service supply of an area (e.g., the ratio of physicians to the population). Following Andersen’s Healthcare Utilization Model, the explanatory variables in this study included self-rated health status, whether or not participants had a chronic disease (such as diabetes mellitus, hypertension, cardiovascular diseases), whether or not they had mobility difficulties (including the ability to squat, bending over; walking from one room to another; going up a flight of stairs safely without help; walk 400 m; locking/unlocking a key using one hand, grab something by hand, raise their hands over their head, and lift something with 4.5 kg with one hand), and whether or not with depression tendency. Depression tendency were measured by the Center for Epidemiologic Studies Depression Scale, CES-D. Individual are considered as having tendency of depression when the CES-D score is 10 and over. These variables are needs-related factors. The predisposing factors included age, marital status (has the individual ever been married), educational level, and doing or not doing any exercise in the past 2 weeks (also a proxy for health beliefs, which was included in predisposing factors). The location of resident and log of predicted income were proxies for enabling factors, and number of physicians per 10,000 population was a proxy for the environmental factor.

### Statistical analysis

To ensure the sample was representative of the entire population, individual weights were applied to the data. For the statistical analysis, a weights value was assigned to each case in the data file. This is normally used to make statistics computed for the data more representative of the population. Survey data are often published along with weights for each observation. Sampling weights provided by the Taiwan NHIS group were used to address different probabilities of survey selection and participation in this study. The weights of each individual in Taiwan NHIS were calculated based on the three variables of “gender” “age”, and “sampling stratification in the county” for the population framework. We applied these sampling weights to perform concentration indices and probit regression model analyses using StataSE 13 conindex command [20] and probit procedures.

We used a probit model to analyze data. According to the literature, factors influencing the health care resource utilization of women and men are different [21–25]; thus, we performed all analysis of male and female participants separately to capture potential gender-specific patterns.

## Results

Table 1 presents the sample distribution of all variables related to whether the outpatient service was used in the past 1 month from the two surveys (2005 and 2009). Health care resource utilization decreased from 2005 (53.8%) to 2009 (46.5%). A goodness-of-fit analysis indicated that the difference of outpatient utilization was statistical significant between 2005 and 2009. Moreover, the utilization of outpatient care by women decreased more than that of men over the surveyed period. For the other variables, similar trends were noted; the proportion of outpatient care utilization was less than 50% in 2009. Notably, the proportion of the population with poor self-rated health, chronic disease, mobility difficulty, or depression status was above 50%. Table 1 shows that the population who used outpatient services had a slightly higher income level during these two study periods.

**Table 1:** Descriptive characteristics of the study sample, by outpatient visit and year, n (%)

Variables	2005 Outpatient using		2009 Outpatient using	
Dependent variable	yes	no	yes	no
Outpatient visit^a^	1300 (53.8)	1115 (46.2)	1187 (46.5)	1367 (53.5)
Independent variable				
Gender	*X*^2^ = 0.4222	*p* = 0.516	*X*^2^ = 1.8042	*p* = 0.179
Female	648 (54.5)	541 (45.5)	652 (45.3)	787 (54.7)
Male	652 (53.2)	574 (46.8)	535 (48.0)	580 (52.0)
Marital status	*X*^2^ = 3.0119	*p* = 0.083	*X*^2^ = 0.0402	*p* = 0.841
Married	875 (55.1)	713 (44.9)	764 (46.6)	874 (53.4)
Unmarried	425 (51.4)	402 (48.6)	423 (46.2)	492 (53.8)
Missing number				1
Age group	*X*^2^ = 4.6048	*p* = 0.032	*X*^2^ = 5.2597	*p* = 0.022
65–74	795 (52.2)	729 (47.8)	679 (44.6)	843 (55.4)
75+	505 (56.7)	386 (43.3)	508 (49.2)	524 (50.8)
Education Level	*X*^2^ = 3.5482	*p* = 0.170	*X*^2^ = 1.7067	*p* = 0.426
Informal education or illiterate	477 (48.5)	449 (48.5)	362 (47.9)	394 (52.1)
Elementary education	535 (54.8)	442 (45.2)	536 (45.2)	650 (54.8)
Junior high school or above	288 (56.2)	224 (43.8)	284 (47.6)	312 (52.4)
Missing number			5	11
Self-reposted health	*X*^2^ = 60.2724	*p* = 0.000	*X*^2^ = 60.1552	p = 0.000
Poor	918 (59.4)	628 (40.6)	853 (52.0)	788 (48.0)
Fair	249 (47.6)	274 (52.4)	238 (39.9)	358 (60.1)
Good	133 (38.4)	213 (61.6)	96 (30.6)	218 (69.4)
Chronic disease	*X*^2^ = 82.5895	*p =* 0.000	*X*^2^ = 70.7057	*p* = 0.000
With chronic disease	740 (63.4)	428 (36.6)	773 (53.8)	664 (46.2)
Without chronic disease	560 (44.9)	687 (55.1)	414 (37.1)	703 (62.9)
Mobility	*X*^2^ = 50.1088	*p =* 0.000	*X*^2^ = 44.0703	*p =* 0.000
Without mobile difficulty	541 (46.4)	625 (53.6)	528 (40.1)	788 (59.9)
With any mobile difficulty	759 (60.8)	490 (39.2)	659 (53.2)	579 (46.8)
Depress	*X*^2^ = 15.4001	*p =* 0.000	*X*^2^ = 16.4124	*p =* 0.000
CES-D scoring under 10 (without)	997 (51.8)	927 (48.2)	967 (44.8)	1193 (55.2)
CES-D scoring 10 and more (with)	303 (61.7)	188 (38.3)	220 (55.8)	174 (44.2)
Whether or not do exercise in past two weeks	*X*^2^ = 3.1783	*p* = 0.075	*X*^2^ = 1.1819	*p* = 0.277
Not doing any exercise in past two weeks	520 (51.7)	486 (48.3)	551 (45.4)	664 (54.6)
Doing any exercise in past two weeks	780 (55.4)	629 (44.6)	636 (47.5)	703 (52.5)
Location in Taiwan	*X*^2^ = 3.2859	*p* = 0.350	*X*^2^ = 3.3526	*p* = 0.340
Eastern and offshore Islands area	85 (49.1)	88 (50.9)	117 (52.0)	108 (48.0)
Central region	348 (56.2)	271 (43.8)	338 (46.6)	387 (53.4)
Northern	420 (52.8)	375 (47.2)	299 (45.1)	364 (54.9)
Southern	447 (54.0)	381 (46.0)	433 (46.0)	508 (54.0)
Physician no. per 10,000 populations in area^b^	*X*^2^ = 1.8863	*p* = 0.389	*X*^2^ = 2.1937	*p* = 0.334
1	517 (54.4)	434 (45.6)	119 (42.4)	162 (57.6)
2	452 (55.0)	370 (45.0)	581 (46.8)	660 (53.2)
3	331 (51.6)	311 (46.2)	487 (47.2)	545 (52.8)
Lpinco^c^	Mean (s.e)		Mean (s.e)	
	8.94 (0.96)	8.91 (0.99)	9.01 (1.35)	8.99 (1.39)

Note: Outpatient visit: whether the participant had an outpatient visit in the past 1 month

*SE* Standard error

^a^The *p* value of goodness-of-fit test between 2005 and 2009 is less than 0.0001, which is a statistical significant

^b^Ratio: 1 for fewer than 70 physicians, 2 for 71–90 physicians, and 3 for 91 or more physicians

^c^log of equivalent income (TW$, 2009)

Table 2 presents the concentration indices for outpatient utilization. The results show that income-related inequality was slightly in favor of those with a higher income but not statistically significant in both periods among both men and women. For elderly women, the CI decreased from 0.0256 in 2005 to − 0.0098 in 2009; that is, it favored those with a higher income in 2005 and those with a lower income in 2009. For men, the CI exhibited a similar trend, decreasing from 0.0379 in 2005 to 0.0346 in 2009.

**Table 2:** Comparison of the CI of outpatient resource utilization inequality between sexes and between the two surveys

	2005		2009		difference	
	Index value	Stand. error	Index value	Stand. error	Index value (s.e.)	p value
Female	0.0256	0.0334	- 0.0098	0.0304	−0.0354 (0.0452)	0.4328
Male	0.0379	0.0329	0.0310	0.0346	−0.0069 (0.0478)	0.8849

Table 3 presents the contributions to inequality in the proportion of outpatient resource utilization for each survey period for elderly women. For each year, the first three columns list the estimated partial effect obtained from the probit model, the elasticity of outpatient resource utilization for every explanatory variable, and each regressor’s CI, respectively. Elasticity represents the sensitivity of outpatient visit utilization to alterations in the covariate. A positive elasticity indicates that the individual with this characteristic has a higher likelihood of using outpatient services. The third column presents the partial CI (also corrected by Erreygers’ normalization) for every determinant. A positive (negative) sign means that the variable has a distribution that favors those with a higher (lower) income. For example, factors such as being aged between 65 and 74 years, education level (elementary school, junior high school or above), and self-reported good health among women had positive CIs, indicating that these factors were concentrated among older women with higher incomes. The absolute contribution of every factor to overall inequality with regard to income is presented in column four. It is obtained using the elasticity and partial CI for every factor. The absolute contribution depends on both the effect of every variable on outpatient visit utilization and its unequal distribution based on income. A negative (positive) absolute contribution of an explanatory variable indicates that, if inequality related to outpatient visits is determined by only that variable, it would favor those with a lower (higher) income. In other words, a negative (positive) value for the absolute contribution of explanatory variables means that the inequality in proportional outpatient resource utilization would increase (decrease) if that variable were to become equally distributed across the distribution of wealth.

**Table 3:** Probit regression and contributions to inequality in outpatient visit in 2005 and 2009, women

variable	2005				2009			
	coefficient	elasticity	Corrected CI	contribution	coefficient	elasticity	Corrected CI	contribution
Lpinco	0.1029	0.3402	0.0353	0.0480	0.7628**	2.5991	0.0322	0.3249
Age group (ref: 75+)								
65–74	0.0036	0.0009	0.0385	0.0001	- 0.0224	- 0.0054	0.1017	- 0.0022
Marital status (ref = unmarried)								
Married	0.0566	0.0115	0.0469	0.0022	- 0.0911	- 0.0181	0.0413	- 0.0030
Education Level (Ref = informal education or illiterate)								
Elementary education	0.1389	0.0175	0.3431	0.0240	- 0.5489*	- 0.0849	0.2286	- 0.0777
Junior high school or above	0.2830	0.0128	0.8360	0.0427	- 0.9739*	- 0.0650	0.8213	- 0.2137
Self-reposted health (Ref = fair)								
Good	- 0.0289	- 0.0013	0.1397	- 0.0007	0.0204	0.0010	0.2550	0.0010
Bad	0.1137	0.0291	- 0.0523	- 0.0061	0.3136**	0.0772	- 0.0597	- 0.0184
Chronic disease (Ref = without)								
With chronic disease	0.3101***	0.0604	0.0206	0.0050	0.3242***	0.0730	- 0.0264	- 0.0077
Mobility (Ref = without mobile difficulty)								
With any mobile difficulty	0.3011**	0.0734	- 0.0741	- 0.0217	0.1119	0.0237	- 0.0767	- 0.0073
Depress (Ref = without, CES-D scoring under 10)								
With	0.2190*	0.0189	- 0.1697	- 0.0128	0.1929	0.0122	- 0.1384	- 0.0067
Whether or not dosing exercise in past two weeks (Ref = not doing)								
Doing any exercise	0.1374	0.0295	0.0696	0.0082	0.0985	0.0193	0.0760	0.0059
Location in Taiwan (Ref = Central region)								
Eastern and offshore Islands	- 0.1872	- 0.0021	- 0.4137	0.0035	0.2135	0.0024	- 0.1348	- 0.0013
Northern	- 0.2156	- 0.0304	0.3498	- 0.0426	- 0.02547	- 0.0039	0.2133	- 0.0033
Southern	- 0.0564	- 0.0067	- 0.1367	0.0036	0.0505	0.0058	- 0.1270	- 0.0029
Physician numbers per 10,000 populations in area^a^ (Ref = 2)								
1	0.1047	0.0142	−0.1461	−0.0083	0.0549	0.0036	0.1100	0.0016
3	−0.1118	−0.0126	0.3999	−0.0202	−0.0610	−0.0104	0.1438	−0.0060

Outpatient visit: whether the participant had an outpatient visit in the past 1 month

Ref: reference group

Lpinco: log of equivalent income (TW$, 2009)

^a^Ratio: 1 for fewer than 70 physicians, 2 for 71–90 physicians, and 3 for 91 or more physicians

*Statistically significant at the 95% level (*p* < 0.05);

**Statistically significant at the 99% level (*p* < 0.01)

*** Statistically significant at the 99.9% level (*p* < 0.001)

The partial effects shown in Table 3 indicate that older women with chronic diseases, mobility difficulties, and a tendency toward depression (CES-D score ≧ 10) showed a significantly higher probability of outpatient resource utilization compared with older women without these complications in 2005. In 2009, older women with a higher income, self-reported poor health, chronic diseases, and tendency toward depression were significantly and positively associated with outpatient resource utilization. Having a higher education level reduced the probability of outpatient resource utilization. Income only had a significant and positive association with outpatient resource utilization in 2009.

In Table 3, positive (negative) signs for the CCIs demonstrate that the explanatory variables had distributions that favored those with a higher (lower) income. Most variables demonstrated the expected sign, for example poor self-reported health with a higher outpatient utilization proportion. The contribution of income increased from 0.00480 to 0.3249, as shown in Table 3, indicating that, despite the decrease of inequality during the financial crisis, its proportion in terms of total inequality increased. This finding can be attributed to the higher elasticity of outpatient visit utilization when compared with the incomes of elderly women in 2009.

Table 4 presents the contributions to inequality in relation to outpatient service utilization for each survey among older men. The partial effects presented in Table 4 suggest that married older men with a higher education, self-reported poor health, chronic diseases, and any mobility difficulty were significantly and positively associated with outpatient resource utilization in 2005. Conversely, living in eastern and offshore island areas reduced the probability of outpatient resource utilization. Older adults with chronic diseases and mobility difficulties exhibited a significantly higher probability of clinical utilization than those without these complications among older women in 2009. The positive (negative) signs for CCIs in Table 4 point toward the explanatory variables having a distribution favoring those with high (low) incomes. Notably, there were changes in these signs of regression coefficient for men with chronic diseases: the pro-high-income distribution (0.0495) in 2005 shifted to a pro-low-income distribution (− 0.0144) in 2009. Furthermore, for areas where the ratio of physicians to the population was under 70 physicians per 10,000 population: the pro-low-income (− 0.1353) distribution changed to a pro-high-income (0.1118) distribution during the financial crisis. The increase in the contribution of income from − 0.0476 to 0.0142, as shown in Table 4, indicates that its proportional influence on total inequality had increased despite the decrease in inequality during the financial crisis.

**Table 4:** Probit regression and contributions to inequality in outpatient visit in 2005 and 2009, men

variable	2005				2009			
	coefficient	elasticity	Corrected CI	contribution	coefficient	elasticity	Corrected CI	contribution
Lpinco	- 0.0758	- 0.2562	0.0464	- 0.0476	0.0159	0.0559	0.0634	0.0142
Age group (ref = 75+)								
65–74	- 0.2248*	- 0.0491	- 0.0045	0.0009	- 0.1445	- 0.0320	- 0.0733	0.0094
Marital status (Ref = unmarried)								
Married	0.2970**	0.0858	0.0343	0.0118	0.2039	0.0611	0.0204	0.0050
Education Level (ref: informal education or illiterate)								
Elementary education	0.3877**	0.0648	- 0.1468	- 0.0380	- 0.0517	- 0.0093	- 0.3138	0.0117
Junior high school or above	0.4486**	0.0554	0.6494	0.1440	0.0077	0.0012	0.5377	0.0026
Self-reposted health (ref: fair)								
Good	- 0.1742	- 0.0109	0.1653	- 0.0072	- 0.2401	- 0.0132	0.1157	- 0.0061
Bad	0.2450*	0.0542	- 0.0759	- 0.0165	- 0.0450	- 0.0097	- 0.0736	0.0029
Chronic disease (Ref = without)								
With chronic disease	0.4884***	0.0795	0.0495	0.0158	0.2321*	0.0446	- 0.0144	- 0.0026
Mobility (ref: without mobile difficulty)								
With any mobile difficulty	0.2982**	0.0442	- 0.1109	- 0.0196	0.4926***	0.0652	- 0.0519	- 0.0135
Depress (ref: without, CES-D scoring under 10								
With	0.0571	0.0035	- 0.1278	- 0.0018	0.1264	0.0059	- 0.1216	- 0.0029
Do any exercise in past two weeks (ref: not)								
Doing any exercise	0.0309	0.0071	0.0856	0.0024	0.1562	0.0342	0.0910	0.0125
Location in Taiwan (ref: Central region)								
Eastern and offshore Islands	- 0.5468**	- 0.0065	- 0.3199	0.0083	0.1906	0.0022	- 0.1516	- 0.0013
Northern	- 0.0741	- 0.0111	0.1878	- 0.0084	0.0846	0.0133	0.1880	0.0100
Southern	- 0.2021	- 0.0214	- 0.0920	0.0079	0.0178	0.0020	- 0.0836	- 0.0007
Physician numbers per 10,000 populations in area ^a^ (Ref = 2)								
1	−0.2362*	−0.0298	- 0.1353	0.0161	0.0361	0.0024	0.1118	0.0011
3	−0.2903**	−0.0322	0.3065	- 0.0394	0.1137	0.0196	0.1275	0.0100

Outpatient visit: whether the participant had an outpatient visit in the past 1 month

Ref: reference group

Lpinco: log of equivalent income (TW$, 2009)

^a^Ratio: 1 for fewer than 70 physicians; 2 for 71–90 physicians; 3 for 91 or more physicians

*Statistically significant at the 95% level (p < 0.05);

**Statistically significant at the 99% level (p < 0.01);

*** Statistically significant at the 99.9% level (p < 0.001)

## Discussion

The results reveal that income-related inequality in the use of outpatient services was not statistically significant from 2005 to 2009 among older adults in Taiwan. This implies that the NHI can protect older people from economic crises related to medical resource utilization.

For older women, the effect of income on outpatient resource utilization was statistically significant in 2009, even though income-related inequality in outpatient visits had not changed significantly across the study’s duration. The higher the income of a person was, the higher was the proportion of their outpatient resource utilization. The CI distribution favored women with higher incomes in 2009. This result may be explained by the fact that women are more vulnerable than men to external economic crises, meaning their income significantly affects on outpatient resource utilization after an economic crisis. Moreover, this result is similar to those of related studies. Recently, Mosquera et al. explored the impact of household income on adolescents’ medical resource utilization. Their results indicated that women in the lowest two quintiles of household income and who were older had more clinical visits than others in higher-income quintiles. However, household income did not significantly affect clinical visits in the male sample [26].

The needs variables had the most significant effect on the use of outpatient resources among all other confounding variables in both the male and female groups. This result is consistent with the needs part of the Andersen model and the same as those of other studies [18, 24, 27].

In the present study, women with a lower education level were found to have a significantly greater probability of outpatient resource utilization in 2009. In contrast, men with a higher education level had a significantly higher probability of outpatient resource utilization probability in 2005, although their education level had no significant effect on outpatient resource utilization in 2009. The effects of education level on health care resource utilization are inconsistent in numerous studies, which showed that a higher education level results in either higher medical resource utilization [28, 29], or higher education level results in lower medical resource utilization [30], and that no significant relationship exists between education level and health care resource utilization [31]. Our results indicate that national health insurance can protect vulnerable members of society in terms of medical care utilization after a global economic crisis.

We used two area variables, namely resident location (as a proxy for enabling factors) and the ratio of physicians per 10,000 population (as a proxy for the health care resource environment). The enabling environment factor covered variables such as degree of urbanization and regional economic situation. Resident location had a significant effect on outpatient resource utilization among older adults in 2005; however, this effect was not significant in 2009. This could be interpreted as indicating that the economic situation differed across Taiwan in 2005. The eastern areas of Taiwan and its offshore islands had a lower economic status in 2005, and residents living in these two areas had a lower utilization rate. Most studies have shown that people located in urban regions have a higher probability of using health care services [18, 19]. Wang et al. found that location of residence contributed more than individual income in determining access to health care. Quashie and Pothisiri also demonstrated that older adults living in rural areas had a significantly higher probability of health care resource utilization. Our results provide some support for previous findings, for example that in 2005, men living in Taiwan’s east or offshore islands had a lower probability of using outpatient health care resources than others. However, Taiwan’s economy was affected by the economic crisis of 2007–2008, which may have reduced the difference in health care resource utilization based on the economic situations in different areas. As such, location of residence did not significantly affect medical utilization among men in 2009. Men living in areas with a lower ratio of physicians to the population or those living in an area with a higher ratio of physicians to the population had lower outpatient resource utilization. This may be explained by the nonlinear relationship between number of physicians and medical resource utilization [32, 33].

Our results show that income-related inequality in outpatient service utilization did not significantly favor those older adults with lower incomes from 2005 to 2009.

Although overall income-related inequality with regard to health care resource utilization decreased slightly during the study period, we found that income had a positive and significant effect on outpatient resource utilization among women in 2009, and the absolute contribution of income to inequality increased from 0.0480 in 2005 to 0.3249 in 2009. For men, income did not significantly affect medical resource utilization in 2005 and 2009. However, the corrected CI and absolute contribution increased from 0.0464 and − 0.0476 in 2005 to 0.0634 and 0.0142 in 2009, respectively. This suggests that the financial crisis increased the influence of income-related inequality on outpatient care utilization, especially among women.

The correlation between income-related inequality and medical resource utilization is inconsistent. Most studies have shown that outpatient care utilization favors those with a lower income and that the utilization of other forms of medical care (e.g., oral health care, specialist care, inpatient care) favors those with a higher income [27, 34].

This study had several limitations. The estimated income variable may have introduced bias, and we could not predict the direction in which inequality was affected by potential biases. Employing additional potential relevant variables such as personal wealth, information on which was not available in our secondary dataset, would help to enhance the predictive power of the income variable. Nevertheless, despite these areas for improvement, our analysis provided valuable information regarding income-related inequality and medical care utilization among older adults during the 2007–2008 financial crisis in Taiwan.

## Conclusions

Taiwan’s NHI guarantees accessible health care to older adults and maintains the income-related equality of medical care utilization among this group. This situation is aligned with the World Bank’s public insurance pillar that promotes protecting the economic security of older adults. However, after controlling other influencing factors, the present study revealed that for women, the contribution of income to absolute inequality increased during the period of the financial crisis. For countries with aging populations, this study provides a reference by using Taiwan’s NHI as an example of the achievement of economic security for older adults in relation to health care resource utilization. The results indicate that particular attention should be paid to the vulnerability of women in terms of economic security during such crises.

## Data Availability

The data that support the findings of this study are available from the National Health Research Institutes (NHRI), Taiwan (website: http://nhis.nhri.org.tw/2009nhis.html; http://nhis.nhri.org.tw/2005nhis.html). However restrictions apply to the availability of these data, which were used under license for the current study, and so are not publicly available. Data are however available from the corresponding author upon reasonable request and with permission of NHRI, Taiwan.

## Abbreviations

CCIs: Corrected concentration indices
CI: Concentration index
NHI: National Health Insurance
NHIS: National Health Interview Survey
NT$: New Taiwan Dollars
